# A DAG-Based Offloading Strategy with Dynamic Parallel Factor Adjustment for Edge Computing in IoV

**DOI:** 10.3390/s25196198

**Published:** 2025-10-06

**Authors:** Wenyang Guan, Qi Zheng, Xiaoqin Lian, Chao Gao

**Affiliations:** School of Computer and Artificial Intelligence, Beijing Technology and Business University, Beijing 100048, China; 20151005@btbu.edu.cn (W.G.);

**Keywords:** Internet of Vehicles, mobile edge computing, task offloading, directed acyclic graph, dynamic adjustment

## Abstract

With the rapid development of Internet of Vehicles (IoV) technology, massive data are continuously integrated into intelligent transportation systems, making efficient computing resource allocation a critical challenge for enhancing network performance. Due to the dynamic and real-time characteristics of IoV tasks, existing static offloading strategies fail to effectively cope with the complexity caused by network fluctuations and vehicle mobility. To address this issue, this paper proposes a task offloading algorithm based on the dynamic adjustment of the parallel factor in directed acyclic graphs (DAG), referred to as Dynamic adjustment of Parallel Factor (DPF). By leveraging edge computing, the proposed algorithm adaptively adjusts the parallel factor according to the dependency relationships among subtasks in the DAG, thereby optimizing resource utilization and reducing task completion time. In addition, the algorithm continuously monitors network conditions and vehicle states to dynamically schedule and offload tasks according to real-time system requirements. Compared with traditional static strategies, the proposed method not only significantly reduces task delay but also improves task success rates and overall system efficiency. Extensive simulation experiments conducted under three different task load conditions demonstrate the superior performance of the proposed algorithm. In particular, under high-load scenarios, the DPF algorithm achieves markedly better task completion times and resource utilization compared to existing methods.

## 1. Introduction

With the rapid development of the Internet of Vehicles (IoV) and intelligent transportation systems, the demand for communication and computation in vehicles has grown explosively [1]. As an integral part of next-generation intelligent transportation, IoV enables real-time information exchange through vehicle-to-vehicle (V2V), vehicle-to-infrastructure (V2I), and vehicle-to-cloud (V2C) communications. In this process, vehicles are not only participants in the transportation system but also act as both data producers and consumers. However, due to the limited computing capabilities of onboard devices, it is difficult to independently handle computation-intensive tasks such as autonomous driving decision-making, route planning, video analysis, and fleet coordination [2]. Therefore, how to allocate computing resources efficiently and guarantee low-latency task execution in highly dynamic vehicular environments has become a pressing challenge.

Cloud computing has been widely employed in IoV-related applications to support task processing [3]. Nevertheless, its centralized architecture results in long physical distances between cloud servers and vehicles, introducing significant transmission latency and making it unsuitable for latency-sensitive tasks. In contrast, edge computing pushes computation and storage resources closer to vehicles, for example, at roadside units (RSUs), thereby reducing latency, alleviating bandwidth pressure, and improving computational efficiency [4,5]. Studies have demonstrated that tasks such as real-time route planning [6] and obstacle detection [7] can be offloaded to edge nodes via RSUs, which alleviates the computational burden on vehicles and improves both responsiveness and reliability [8,9].

Against this background, task offloading has emerged as a crucial research direction in IoV. An effective task offloading strategy can significantly reduce the computational load of vehicles while leveraging the powerful computing capabilities of edge servers to shorten execution time and improve service quality [10]. However, task offloading in IoV faces multiple challenges. On one hand, IoV environments are highly dynamic, with rapidly changing topologies due to vehicular mobility, which complicates task scheduling. On the other hand, vehicular tasks are heterogeneous and often subject to strict delay constraints, particularly in autonomous driving scenarios where tasks such as route planning and obstacle detection require real-time execution [11]. Moreover, factors such as network latency, the heterogeneous computing capacity of edge nodes, and resource contention significantly affect the performance of offloading strategies [12,13,14].

As vehicular applications become more sophisticated, the size and structure of generated tasks are also increasing in complexity. A promising approach to address this challenge is to decompose large tasks into subtasks for more efficient scheduling and execution. In this regard, Directed Acyclic Graph (DAG)-based task decomposition models have been widely adopted [15,16,17]. The DAG model explicitly represents task dependencies: dependent tasks are executed sequentially, while independent tasks can be executed in parallel, thus improving both resource utilization and scheduling flexibility.

In DAG-based models, the parallel factor is a critical parameter that determines the number of subtasks that can be executed concurrently. An appropriately set parallel factor can maximize concurrency while satisfying task dependency constraints, thereby enhancing resource utilization [18]. However, setting the parallel factor too high leads to excessive resource contention and longer completion times, whereas setting it too low results in underutilization of computing resources. Recent studies have indicated that in highly dynamic IoV environments, DAG-based task offloading combined with dynamic parallel factor adjustment can significantly improve system performance [19]. For instance, when network load is low, the system can increase the parallel factor to accelerate execution, whereas when resources are heavily utilized, the parallel factor can be reduced to alleviate contention and optimize latency.

Motivated by these insights, this paper proposes a Dynamic adjustment of Parallel Factor (DPF) algorithm. The algorithm introduces a dynamic adjustment mechanism into DAG-based task offloading to adaptively regulate concurrency while maintaining task dependencies. By doing so, the DPF algorithm improves execution efficiency, enhances resource utilization, and reduces overall delay under constrained edge computing resources. Experimental results demonstrate that the proposed algorithm achieves excellent performance under various network conditions and effectively addresses the challenges of high mobility and resource contention in IoV.

## 2. Related Work

With the development of Internet of Vehicles (IoV) technology, researchers have conducted in-depth explorations of the applications of edge computing in IoV environments. The primary characteristics of IoV are its dynamic nature and real-time requirements, which pose new challenges to computing architectures. To address these challenges, many studies have focused on optimizing task offloading and resource management strategies.

### 2.1. Offloading Scenario

MEC offloading can be categorized into two primary scenarios: single-server multi-user and multi-server multi-user. For the first scenario, Singh et al. [20] developed an energy-efficient task offloading strategy (EETOS) based on the Levy-flight moth flame optimization (LMFO) algorithm. This strategy aims to minimize energy consumption and end-to-end delay for IoT sensor applications in fog-cloud computing systems. Li et al. [3] proposed an adaptive transmission strategy based on cloud computing for task offloading and transmission in an IoV architecture. By dynamically assigning tasks to different cloud link lists and considering node characteristics for distributed processing, this strategy optimizes transmission delay and resource utilization. Ali et al. [21] introduced a novel task scheduling algorithm that addresses energy consumption and execution time issues in mobile devices through an energy-efficient dynamic decision-making approach. This model rapidly adapts to cloud computing tasks while optimizing energy and time computations for mobile devices.

For the second scenario, Xu et al. [9] proposed a cloud computing offloading strategy based on a multi-strategy cooperation-seal optimization algorithm (M-TSA). This strategy integrates task priority and computational offloading node prediction, simulating vehicle movement under real-world road conditions to optimize task offloading delay, energy consumption, and efficiency. Shu et al. [22] introduced an EFO algorithm-based offloading scheme for multi-user edge computing systems, which efficiently offloads the most suitable IoT tasks or subtasks to edge servers to minimize the expected execution time. Shao et al. [23] proposed a dynamic edge-end computing collaboration architecture for urban IoV, offering a more flexible and adaptive task allocation approach. In this architecture, edge nodes and vehicle terminals collaborate to process tasks. The paper considers task delay and overhead, task transmission models, task priority, and the computational capacities of edge nodes and vehicle terminals. It defines task utility and formulates the task allocation problem as an optimization model.

However, none of the above studies have addressed collaborative computation among edge servers. If effectively utilized, inter-server coordination could further enhance the performance of offloading optimization.

### 2.2. Task Model

In the current research, tasks are classified into two types: indivisible tasks and divisible tasks.

For indivisible tasks, Zhu et al. [24] proposed a task offloading strategy based on cloud-fog collaborative computing. This strategy introduces a vehicle-to-vehicle (V2V)-assisted task forwarding mechanism and designs a forwarding vehicle prediction algorithm based on environmental information. Additionally, a multi-strategy improved genetic algorithm (MSI-GA) is proposed, which initializes populations using a chaotic sequence, optimizes adaptive operators by comprehensively considering influencing factors, and incorporates Gaussian perturbation to enhance the local optimization capability of the algorithm. Plachy et al. [25] proposed a low-complexity computing and communication resource allocation method for offloading real-time computational tasks generated by mobile users. This method utilizes probabilistic modeling of user mobility to pre-allocate computing resources at base stations and selects appropriate communication paths between users and base stations with pre-allocated computing resources.

For divisible tasks, Du et al. [26] proposed a blockchain-based directed acyclic graph (DAG) structure for secure and efficient information sharing in IoV. The paper also designed a driving decision-based tip selection algorithm (DDB-TSA) and a reputation-based rate control strategy (RBRCS) to enhance information-sharing security. Yan et al. [27] proposed an MEC network comprising two users, where each wireless device (WD) has a series of tasks to execute. Considering task dependencies between the two WDs—where one WD’s task input requires the final output of the other—the paper investigates the optimal task offloading strategy and resource allocation (e.g., offloading transmission power and local CPU frequency) to minimize the weighted sum of WDs’ energy consumption and task execution time.

Our work further extends divisible task research by considering dependencies among subtasks and exploring offloading parallel to enhance system utilization.

### 2.3. Offloading Strategy

Dai et al. [28] proposed a computation offloading scheme based on deep reinforcement learning (DRL). This scheme employs a deep Q-network (DQN) to adapt to the dynamic vehicular edge computing environment, quickly learning offloading decisions by balancing the exploration and exploitation process to minimize the average task processing delay. Sun et al. [29] designed an Adaptive Learning Task Offloading (ALTO) algorithm based on the multi-armed bandit theory to minimize the average offloading delay. ALTO operates in a distributed manner without requiring frequent state exchanges and incorporates input awareness and occurrence awareness to adapt to dynamic environments. Misra et al. [30] proposed a three-tier architecture to address task offloading in a mobile cloud environment. This architecture, named “Selection of Best Destination to Offload,” attempts to offload tasks first to nearby mobile devices and edge cloud servers before considering remote cloud servers. The first two tiers consist of nearby mobile devices and edge cloud servers, while the third tier comprises remote cloud servers.

In summary, resource allocation in the task offloading process is a critical research direction in vehicular networks. The computing, storage, and network bandwidth resources of edge computing nodes are limited. Therefore, when handling the large number of tasks generated by vehicles, efficient resource allocation is crucial [31]. Studies have shown that optimizing resource allocation strategies can not only enhance task processing efficiency but also significantly reduce task transmission latency and system energy consumption [32]. To achieve efficient resource allocation, the system must monitor network conditions, task loads, and the resource utilization of edge nodes in real-time [33]. Based on this information, the system can dynamically adjust task offloading decisions and resource allocation strategies to maximize task success rates and resource utilization efficiency under different scenarios [34]. Furthermore, resource allocation must consider vehicle mobility, as high-speed movement may disrupt communication links with edge nodes. Therefore, offloading strategies must be highly robust and flexible [35].

Although significant progress has been made in vehicular networks and edge computing research, challenges remain in achieving efficient task offloading and resource allocation in dynamic environments. Building on existing studies, this paper proposes a novel dynamic task offloading algorithm to further enhance system performance in vehicular networks.

## 3. System Model

In this section, we will provide a detailed introduction to the network model, task model, communication model and present our optimization problem.

### 3.1. Network Model

**Figure 1:**
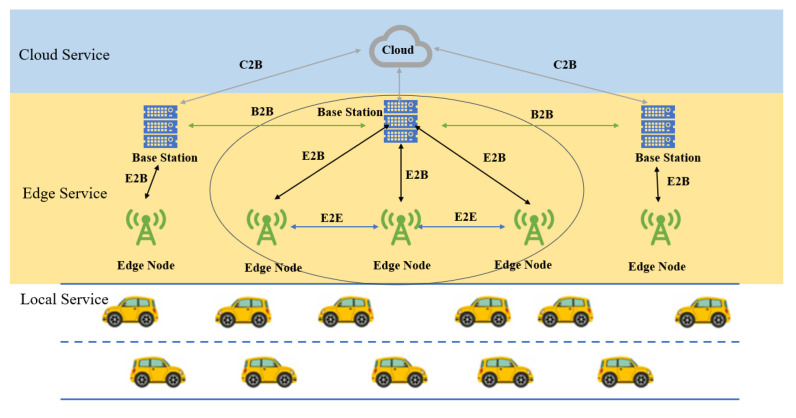
Overall framework of task offloading in vehicular networks based on edge computing.

This paper proposes a vehicular task offloading system that integrates cloud computing and edge computing, as shown in Figure 1, featuring a three-layer network architecture. Vehicles act as task initiators and can send offloading requests to the cloud via the nearest edge node. The cloud then generates an offloading strategy and assigns tasks to target nodes, which may include the vehicle’s local processor, edge nodes, base stations, or cloud servers. Once the task is completed, the execution results are returned to the vehicle.

**Figure 2:**
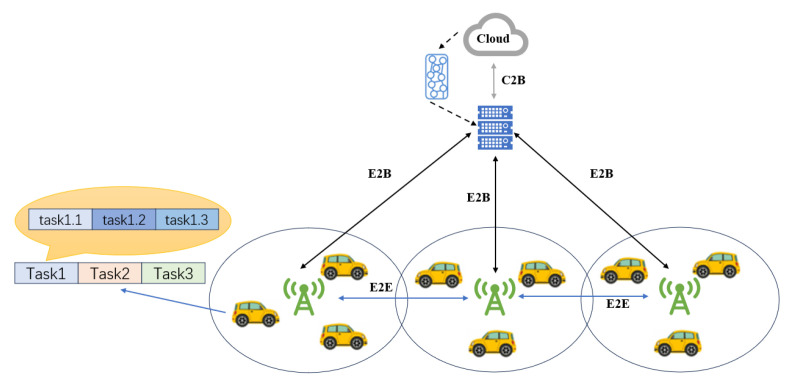
Offloading framework based on subtask directed acyclic.

The edge computing layer consists of multiple edge nodes. As shown in Figure 2, every three adjacent edge nodes form a small-scale network cluster, and a base station monitors the status of the nodes and manages task scheduling within the cluster. Base stations are connected to edge nodes via optical fiber, and the cloud server monitors the operational status of all base stations to enable inter-cluster scheduling. As edge nodes, base stations, and the cloud server can communicate via optical fiber, vehicles are considered capable of offloading subtasks to any available server across the network, ensuring efficient task processing.

### 3.2. Communication Model

The edge server layer consists of a collection of edge nodes and base stations. Each vehicle has a set of tasks S={1,2,…,j,…,s} to be executed. The execution process of the *j*-th task on vehicle *i* is modeled as a Directed Acyclic Graph (DAG), denoted as $G_{i}^{j}=\left(V_{i}^{j},E_{i}^{j}\right)$, where $V_{i}^{j}$ represents the set of subtasks of task *j*, and $E_{i}^{j}$ denotes the dependency relationships among these subtasks. It is assumed that each task can be decomposed into subtasks according to computational requirements, with each subtask representing the smallest unit of computation. Subtasks can either be executed locally on the vehicle or offloaded to any available server in the network, depending on the offloading strategy.

For local execution, assuming the vehicle has a computational capability of $f_{i}$, the execution time of subtask $T_{i,j}^{k}$ on vehicle *i* is given by:

(1) $$t_{i,j}^{k}=\frac{c_{i,j}^{k}}{f_{i}}.$$

The corresponding energy consumption for local execution is:

(2) $$e_{i,j}^{k}=c_{i,j}^{k}\times \delta_{i},$$

where $\delta_{i}$ denotes the energy consumption per CPU cycle for the onboard processor of vehicle *i*.

In this paper, it is assumed that servers of the same type have identical computing capabilities, while different types differ. Let $f=\{f_{e},f_{b},f_{c}\}$ represent the computational capacities of edge servers, base stations, and cloud servers, respectively.

For subtasks offloaded to servers, the total processing time is expressed as:

(3) $$w_{i,j}^{k}=\frac{c_{i,j}^{k}}{f}.$$

(4) $$t_{i,j}^{k^{\prime }}=\frac{d_{i,j}^{k}}{r_{i,e}}+t_{i,j}^{k,\mathrm{Queue}}+w_{i,j}^{k}.$$

where $w_{i,j}^{k}$ is the computation time on the target server, $r_{i,e}$ is the transmission rate between vehicle *i* and the selected server, and $t_{i,j}^{k,\mathrm{Queue}}$ is the queuing delay at the server.

Since edge and cloud servers are assumed to be continuously powered, only the energy consumption at the vehicle side during the offloading process is considered:

(5) $$e_{i,j}^{k^{\prime }}=\frac{q_{i}\times d_{i,j}^{k}}{r_{i,n}},$$

where $q_{i}$ represents the transmission power of vehicle *i*.

### 3.3. Problem Formulation

The ultimate goal of our proposed model is to minimize the average task processing time for all vehicles, thereby meeting stringent delay requirements. To achieve this, we first define the AST of subtask *k* offloaded to server *l*, denoted as ${\mathrm{AST}}_{i,j}\left(k,l\right)$, as follows:

(6) $$\begin{array}{cc} {AST}_{i,j}\left(k,l\right)= & \max\{avail\left\{0\cup \left[l\right]\right\},\max_{k^{\prime }\in pred\left(k\right)}AET\left(k^{\prime }\right)+C_{kk^{\prime }}\}. \end{array}$$

Here, pred(k) represents the set of immediate predecessor subtasks of subtask *k*, and $C_{kk^{\prime }}$ denotes the communication cost between subtask *k* and its predecessor $k^{\prime }$. The term avail{0∪[l]} refers to the earliest available time for the computing resource, which can either be the vehicle itself (local execution) or the target server *l*.

Taking into account the queuing delay and execution order, the AET of subtask *k*, denoted as ${AET}_{i,j}\left(k\right)$, is defined as:

(7) $${AET}_{i,j}\left(k\right)=\min\left(w_{k}^{l^{\prime }}+{AST}_{i,j}\left(k^{\prime },l^{\prime }\right)\right)$$

where $w_{k}^{l^{\prime }}$ is the execution time of subtask *k* on the computing node $l^{\prime }$, which could be either a server or the vehicle itself. The actual completion time of the subtask is determined once all of its predecessors have completed execution and required resources become available.

## 4. Dynamic Adjustment of Parallel Factor Offloading Strategy

**Figure 3:**
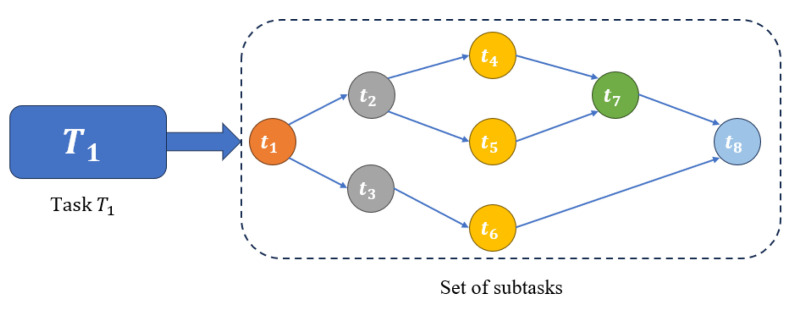
Illustration of task decomposition and subtask dependencies based on a directed acyclic graph.

In this section, we propose DPF, an efficient DAG-based task offloading algorithm that dynamically selects target servers for subtasks. A task is modeled as a DAG and decomposed into multiple subtasks with dependency relations, as shown in Figure 3. For example, task $T_{1}$ represents a complete vehicular task, which is further divided into a set of subtasks $\left\{t_{1},t_{2},\ldots ,t_{8}\right\}$. $t_{1}$ must be executed first; $t_{2}$ and $t_{3}$ can then run in parallel, while tasks $t_{4}$, $t_{5}$, and $t_{6}$ are processed only after the completion of $t_{2}$ and $t_{3}$, respectively. Subtask $t_{7}$ depends on $t_{4}$ and $t_{5}$, and $t_{8}$ requires the outputs of $t_{6}$ and $t_{7}$, so the task completes only after all paths are executed. By adjusting the parallel factor, DPF balances computation across servers, reduces waiting time, and minimizes overall processing delay.

### 4.1. Subtask Scheduling and Prioritization

In edge computing scenarios, complex vehicular tasks are typically decomposable into multiple interdependent subtasks. The DAG is utilized to represent these dependencies, guiding the assignment and execution of subtasks across heterogeneous computing nodes, including local devices, edge servers, and cloud servers.

Let a complex task *S* be decomposed into a set of subtasks *T*, and the dependency among these subtasks be represented as a DAG: G=(V,E), where *V* denotes the set of subtasks (each represented as a vertex), and *E* denotes the set of directed edges indicating dependency relations. A directed edge from subtask $T_{k^{\prime }}$ to $T_{k}$ indicates that $T_{k}$ can only be executed after $T_{k^{\prime }}$ has completed.

The execution order of subtasks has a direct impact on the total latency. Therefore, we first model the scheduling order of subtasks. Let ${\mathrm{data}}_{\left(k,k^{\prime }\right)}$ represent the data volume transmitted from subtask $k^{\prime }$ to *k*. The communication delay between these two subtasks can be modeled as:

(8) $$c_{k,k^{\prime }}=\left\{\begin{array}{cc} 0 & \mathrm{if}\;a_{i,k}=a_{i,k^{\prime }},\\ \frac{{\mathrm{data}}_{k,k^{\prime }}}{r_{i,e}\left(a\right)} & \mathrm{otherwise}. \end{array}\right.$$

Here, $a_{\left(i,k\right)}$ denotes the offloading decision for subtask *k*, and $r_{i,e}\left(a\right)$ is the transmission rate between vehicle *i* and the edge server. If both subtasks are assigned to the same server, communication overhead can be ignored.

We then compute the priority of each subtask based on scheduling urgency using a recursive ranking function:

(9) $$rank\left(k\right)=w_{i,k}^{l}+C_{k,k^{\prime }}+rank\left(k^{\prime }\right).$$

While this ranking reflects computation and communication cost, task urgency must also be considered in vehicular networks. We categorize tasks into four urgency levels: high ($u_{h}$), medium ($u_{m}$), low ($u_{l}$), and non-urgent ($u_{n}$). Each task inherits a fixed urgency level, which is uniformly applied to its subtasks.

We define the urgency ratio ${\mathrm{RTR}}_{U}\left(k\right)$ for each subtask as:

(10) $${RTR}_{U}\left(k\right)=\frac{T_{\mathrm{estimated}}\left(k\right)-T_{\mathrm{current}}\left(k\right)}{T_{\mathrm{deadline}}\left(k\right)-T_{\mathrm{current}}\left(k\right)},$$

where $T_{\mathrm{deadline}}\left(k\right)$ is the deadline, $T_{\mathrm{current}}\left(k\right)$ is the current time, and $T_{\mathrm{estimated}}\left(k\right)$ is the estimated completion time for subtask *k*. A larger value of ${RTR}_{U}\left(k\right)$ indicates higher urgency.

To combine the task complexity and urgency, we propose a weighted priority score: where α and β are tunable parameters that control the trade-off between computational priority and urgency level, depending on system-specific performance objectives. By sorting subtasks based on their priority R(k), and intelligently mapping them to suitable computing nodes, our objective is to minimize the overall task completion time. This optimization can be formulated as:

(11) $$\min\sum_{k\in \max R(k)}t_{i,j}^{\prime \;k}.$$

By jointly considering execution time $t_{i,j}^{\prime }\left(k\right)$ and priority R(k), the system can achieve global optimization of task scheduling and offloading decisions, rather than just locally optimizing individual task execution. The DPF algorithm embodies this principle by ensuring that higher-priority tasks, which are more time-sensitive, are preferentially executed or offloaded to computing nodes with lower latency.

### 4.2. Parallel Factor and Load-Aware Scheduling Model

In this section, we introduce the concept of a parallel factor, which refers to the degree of parallel among mutually independent subtasks in a task’s DAG. By adjusting the parallel factor for each task DAG, the number of executable subtasks at a given time can be increased, thereby expanding the pool of offloading candidates and enabling the selection of a more optimal offloading strategy to reduce overall task processing time.

To monitor the computational load of each server node, we propose a theoretical load model. Let the computing capacity of a server node *l* be denoted by $C_{l}$, representing the number of tasks it can process per second. The current utilization of node *l* is defined as:

(12) $$U_{l}=\frac{n_{l}}{C_{l}},$$

where $n_{l}$ is the number of tasks currently being processed. When $U_{l}>1$, the server is considered overloaded, while $U_{l}<1$ indicates underutilization. The system aims to maintain each node’s utilization near 1 by dynamically adjusting the parallel factor.

In practical terms, we define the utilization $U_{l}\left(t\right)$ of node *l* at time *t* as the ratio of its consumed computing resources to its total capacity, based on the subtasks being processed. Let $T_{l}^{k}\left(t\right)$ denote the set of subtasks running on node *l* at time *t*. The utilization can be computed as:

(13) $$U_{l}\left(t\right)=\frac{\sum_{k\in T_{l}^{k}\left(t\right)}C_{k}}{C_{l}},$$

where $C_{k}$ denotes the computational demand of subtask *k* on node *l*. By continuously monitoring $U_{l}\left(t\right)$, the system can dynamically perceive the computational status of each node and adjust the scheduling strategy and parallel factor accordingly.

From Equation (7), we obtain the actual execution end time ${AET}_{i,j}\left(k\right)$ of subtask *k* on server node *l*, which includes both queuing and computation time. The goal of the DPF algorithm is to achieve a relatively balanced task completion time across all computing nodes.

To do this, we first calculate the total task completion time ${TCT}_{l}\left(t\right)$ of server *l* at time *t*:

(14) $${TCT}_{l}\left(t\right)={AET}_{i,j}\left(k^{\prime \prime }\right),$$

where $k^{\prime \prime }$ is the last subtask in node *l*’s execution queue at time *t*. Hence, the time at which this subtask finishes execution reflects the total processing time of node *l*.

We then calculate the average task completion time across all *n* nodes:

(15) $$\bar{TCT(t)}=\frac{1}{n}\sum_{l=1}^{n}{TCT}_{l}\left(t\right).$$

and the variance of completion time among all nodes:

(16) $${TV}^{2}=\frac{1}{n-1}\sum_{l=1}^{n}{\left[{TCT}_{l}\left(t\right)-\bar{TCT(t)}\right]}^{2}.$$

We define the processing cycle time (${PCT}_{K}$) for subtask *k* as the difference in completion time between it and its predecessor subtask $k^{\prime }$:

(17) $${PCT}_{K}={AET}_{i,j}\left(k\right)-{AET}_{i,j}\left(k^{\prime }\right).$$

When a new subtask is offloaded, the increase in a node’s workload is not only the computation time of that subtask, but also the queuing delay resulting from earlier subtasks. Therefore, incorporating ${\mathrm{PCT}}_{K}$ allows for more precise load balancing adjustments. The complete procedure is illustrated in Algorithm 1.

**Algorithm 1:** DPF Algorithm
 **Input**: $G_{i}^{j}=\left(V_{i}^{j},E_{i}^{j}\right)$, $d_{i,j}^{k}$, $c_{i,j}^{k}$, *f*, $T_{\mathrm{deadline}}\left(k\right)$, R(k) **Output**: $a_{i,j}^{k}$**1** Initialize the number of tasks and subtasks for each vehicle and generate the corresponding DAG for each task **2** Define rank(k) by Equation (9)**3** Calculate R(k) by Equation (11)**4** **for** *each new subtask k* **do****5**    **while** l<n **do**
**6**      Offload *k* to the corresponding node based on the DAG
**7**      **if*** $PCT_{k}<AET_{i,j}\left(k^{\prime }\right)$ **and** $TV^{2}<\theta$ ***then**
**8**        Maintain the DAG**9**      **else**
**10**        Unload subtask *k* to min(AET)’s *N*
**11**         **if** $TV^{2}<\theta$ **then**
**12**          Adjust *Q* and modify the DAG**13**        **else**
**14**          Place *k* into nodes where $AET_{i,j}\left(k^{\prime }\right)<\bar{TCT(t)}$**15**        **end if****16**      **end if****17**    The remaining subtasks are sent to the nodes of this offloading scheme**18**    **end while****19** 
**end for**



## 5. Evaluation Analysis

In the simulation scenario, vehicles travel on a straight, bidirectional road. It includes 100 vehicles [36], each randomly generating 10 to 15 tasks, and each task is by default decomposable into 5 to 15 subtasks. The size of the subtasks and the required CPU cycles are randomly generated within the ranges of 500 KB to 1500 KB and 0.2 GHz to 0.3 GHz, respectively. The transmission power is set to 100 mW, background noise to −100 dBm, and wireless channel bandwidth to 20 MHz. The local CPU frequency of the vehicle is 2 GHz, the CPU frequency of the edge service node is 20 GHz [37], the CPU frequency of the base station server is 50 GHz, and the CPU frequency of the cloud server is 200 GHz.

### 5.1. Task Completion Time

Task completion time is one of the most critical performance indicators in edge computing task offloading, It directly reflects the system’s task processing efficiency.

**Figure 4:**
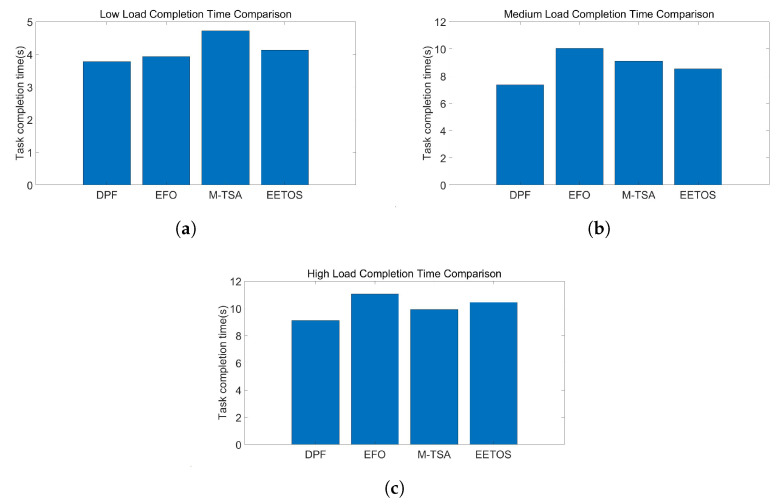
(**a**) Low load completion time. (**b**) Medium load completion time. (**c**) High load completion time.

Figure 4 shows the average task completion time of different algorithms under the three load conditions. In the low-load scenario, all algorithms achieve relatively short completion times due to the abundance of system resources. As the load increases, the advantage of the DPF algorithm becomes more obvious: by decomposing tasks into subtasks and using each node’s computational resources more evenly, the algorithm achieves better load balancing. Compared with the competing methods, DPF reduces task completion time by approximately 0.16 s, 1.17 s, and 0.8 s under low, medium, and high load conditions, respectively. These results demonstrate that DPF can effectively reduce queuing delay by balancing server workloads.

In real IoV scenarios, reducing task completion time by over one second under heavy load is critical for delay-sensitive tasks such as obstacle detection or autonomous driving decisions.

### 5.2. Servers Utilization

Server utilization refers to the ratio between a server’s actual workload and its maximum processing capacity. Ideally, server utilization should be maintained at an appropriate level to maximize the use of server resources while avoiding overload.

**Figure 5:**
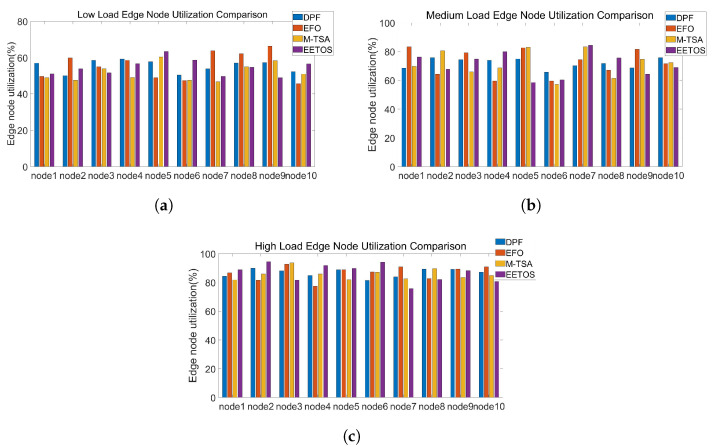
(**a**) Low load node utilization. (**b**) Medium load node utilization. (**c**) High load node utilization.

**Figure 6:**
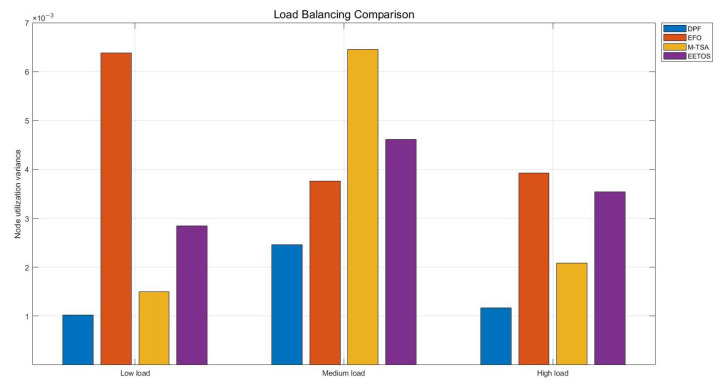
The variance of server node utilization under different loads.

To evaluate server utilization, we monitored the resource usage of ten edge nodes under different task load conditions. Figure 5 presents the server utilization, while Figure 6 shows its variance, reflecting load balancing among nodes. Under high load, the DPF algorithm maintains utilization between 80% and 90%, whereas other algorithms achieve only 65–75%. The utilization variance of DPF is also very low, ranging from 0.001 to 0.003, which is lower than that of the competing algorithm. This is because the DPF algorithm can dynamically adjust the parallel factor, leading to a more balanced use of computational resources across nodes. As a result, each node can achieve its maximum computational potential, thereby avoiding both resource overload and idleness.

In practice, this means DPF maximizes system capacity by keeping all servers consistently well utilized and evenly loaded.

### 5.3. System Scalability

**Figure 7:**
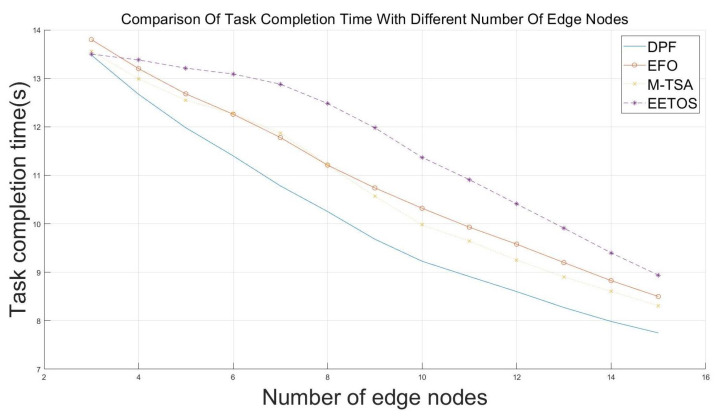
Task completion time under different number of edge nodes.

Due to variations in road length and complexity, the number of deployed edge nodes may differ. To validate the algorithm’s performance under different scenario complexities, we increased the number of nodes from 3 to 15 under high-load task conditions. As shown in Figure 7, when only 3 nodes are available, the difference in task completion time compared to competing algorithm is minimal due to the limited number of available target nodes. As the number of nodes increases, the optimization effect of the DPF algorithm on task completion time becomes more pronounced. With 6 nodes available, the increased number of offloading targets allows the DPF algorithm to demonstrate its advantage in adaptive dynamic adjustment, achieving a task completion time 0.23 s faster than the EFO algorithm. As the number of nodes continues to grow, DPF achieves more rational resource allocation. For 10 or more nodes, the greater number of available offloading targets further enhances the benefits of dynamic adjustment, resulting in a 7.53% performance improvement over M-TSA algorithms, with the optimization trend continuing.

This demonstrates that DPF is applicable to scenarios of varying complexity, with its advantages becoming more prominent as the number of available nodes increases. This indicates that in large-scale vehicular networks, DPF can effectively leverage additional edge nodes to maintain performance improvements as the system scales.

### 5.4. Task Success Rate

Task success rate refers to the proportion of tasks successfully completed within the specified time during the offloading process. A high task success rate indicates that the system can process tasks in a timely manner, avoiding failures caused by insufficient resources or excessive delays. In edge computing systems, the task success rate not only reflects the system’s computational capability but also reveals the effectiveness of resource scheduling. Therefore, it serves as a crucial metric for evaluating system stability and reliability.

**Figure 8:**
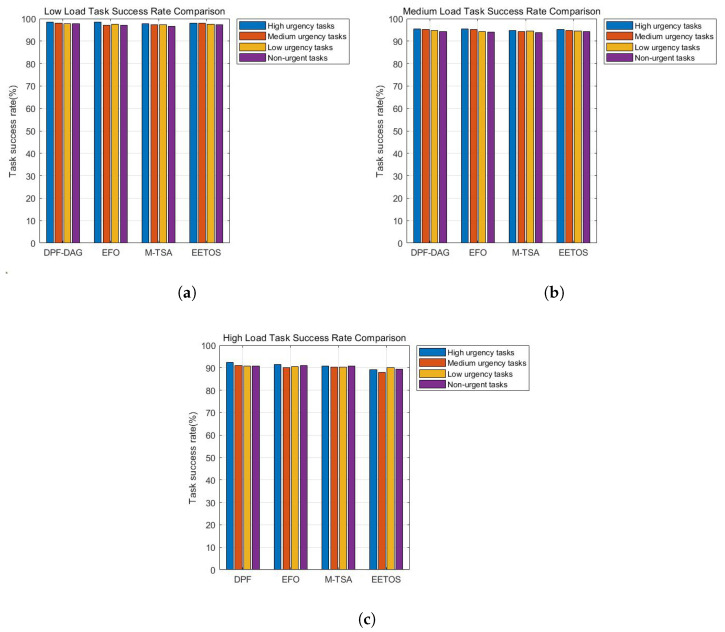
(**a**) Low load task success rate. (**b**) Medium load task success rate. (**c**) High load task success rate.

To evaluate the task success rate, we conducted comparative tests under the four priority levels, with higher-priority tasks typically related to driving safety, requiring preferential processing. The proportion of tasks finished before deadlines is plotted in Figure 8. Across all load levels, DPF consistently provides higher reliability: over 97% at light load, above 94% at medium load, and still above 90% at heavy load. Such improvements come from prioritizing urgent tasks while balancing server workloads, which lowers the risk of deadline violations. This is because the adaptive dynamic adjustment mechanism of the DPF algorithm, which prioritizes urgent tasks while balancing server workloads, thereby reducing the risk of task timeout. This means DPF ensures dependable performance for time-critical vehicular applications even in congested environments.

### 5.5. System Regulation Performance

In a real-world edge computing environment, node instability and external interference are inevitable. Therefore, an algorithm must be able to adapt to fluctuations in computational power. By simulating interference scenarios, we can evaluate the algorithm’s performance under complex conditions, providing both theoretical and empirical support for its practical deployment. This section introduces node interference and examines how the system offloads tasks from constrained nodes to other available nodes, thereby assessing the algorithm’s ability to adjust task distribution and load balancing.

**Figure 9:**
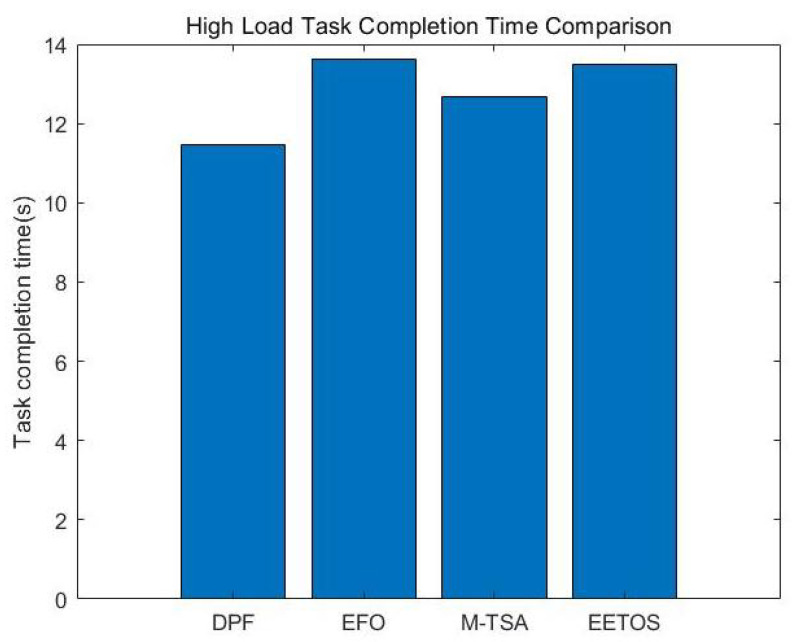
Task Completion Time After Interference.

**Figure 10:**
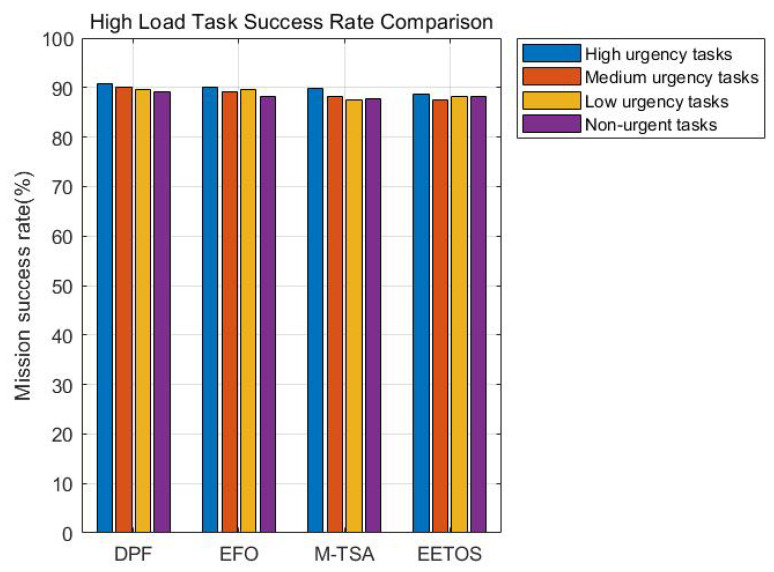
Task Success Rate After Interference.

Figure 9 and Figure 10 illustrate the task completion time and success rate under high load conditions when a node experiences interference. Figure 9, compared with Figure 4c, shows that task completion time increased by 2.23 s after interference. However, it remains 15.92% lower than M-TSA algorithm. Figure 10, compared with Figure 8c, demonstrates that task success rate experienced only a slight drop of 1.45% across four priority levels. Moreover, the system still ensures high-priority tasks receive preferential processing. This is because the DPF algorithm dynamically adjusts the parallel factor and re-offloads subtasks originally assigned to interfered nodes to other available nodes, thereby minimizing the impact of interference to the greatest extent.

From the above evaluation metrics, it can be seen that the DPF algorithm, through dynamic task scheduling and parallel processing optimization, is able to maintain low task completion time while achieving relatively balanced resource utilization in different network environments.

## 6. Conclusions

This paper proposes a DAG based DPF task offloading algorithm to address the challenges of task offloading in IoV environments. The algorithm dynamically adjusts the degree of parallel according to subtask dependencies and server utilization, thereby reducing overall task completion time and enhancing system stability. By modeling the task offloading process under a DAG structure and introducing a dynamic parallel factor adjustment mechanism, this paper provides a flexible and efficient solution for vehicular task scheduling. Simulation results under multiple parameter settings demonstrate that the proposed DPF algorithm outperforms benchmark methods in terms of task completion delay, task success rate, and server utilization. In addition, when certain nodes experience interference, the algorithm is still able to maintain stable performance, which verifies its practicality and robustness in dynamic IoV environments. Nevertheless, this work still has several limitations. The simulation environment assumes relatively balanced computational capabilities between vehicles and servers, whereas real vehicular networks are often highly heterogeneous. The frequent disconnection problem caused by high vehicle mobility has not been explicitly addressed. In addition, the scalability of the algorithm for large-scale or highly complex DAG task graphs requires further validation. These issues will be investigated in our future work.

## Data Availability

Data are contained within the article.
